# Common Buzzards wintering strategies as an effect of weather conditions and geographic barriers

**DOI:** 10.1002/ece3.7793

**Published:** 2021-06-27

**Authors:** Emanuel Stefan Baltag, Istvan Kovacs, Lucian Sfîcă

**Affiliations:** ^1^ Marine Biological Station “Prof. Dr. Ioan Borcea”, Agigea “Alexandru Ioan Cuza” University of Iasi Iasi Iasi Romania; ^2^ Association for Bird and Nature Protection “Milvus Group” Targu Mures Romania; ^3^ Faculty of Geography and Geology “Alexandru Ioan Cuza” University of Iasi Iasi Romania

**Keywords:** Arctic Oscillation, birds of prey, geographic barrier, North Atlantic Oscillation Index

## Abstract

**Aim:**

Migration is a constantly changing adaptation due to the climate condition evolution. The struggle for surviving during harsh winter season is different across Europe, being more complex toward the inner parts of the continent. The current approach explores the Common Buzzard number variation during the cold season and the climatic predictors of birds of prey wintering movements in relation to the possible influences of the Carpathian Mountains, which may act as a geographical barrier providing shelter from cold air outbreak from north and northeast of the continent.

**Location:**

Romania (45°N25°E).

**Taxon:**

Birds of Prey.

**Methods:**

We applied a GLMM to investigate the relation between continental and local climatic factors with the number of Common Buzzard observations in two regions. The first region is located inside the Carpathian Arch and the other one outside, east of this large mountains chain.

**Results:**

The Common Buzzard numbers wintering Eastern from the Carpathian Mountains are highly influenced by AO (*Z* = 2.87, *p* < .05%), while those wintering western are influenced by NAO (*Z* = 2.17, *p* < .05%). This is the first proof of separating influences for biodiversity of AO and NAO at continental scale, outlining the influence limit placed over the Eastern Carpathian Mountains.

**Main conclusions:**

The Carpathian Mountains act like a geographic barrier, separating the wintering Common Buzzard populations from both sides of the mountain range. While the high number of individuals in Moldova is related to their eastern and northeastern Europe origins, in Transylvania the large number of individuals observed is related to the more sheltered characteristics of the region attracting individuals from central Europe. Also, since Transylvania region is well sheltered during cold air outbreak, it represents a more favorable region for wintering. From this point of view, we can consider that the Carpathian Mountains are a geographic barrier for wintering birds of prey.

## Graphical abstract

The analysis of meteoclimatic conditions reveals different spatial origins of Common Buzzard observed in Romania. While the high number of individuals in Moldova is related to their eastern and northeastern Europe origins, in Transylvania the higher number of individuals observed is related to the more sheltered characteristics of the region attracting individuals from central‐eastern Europe. Also, the Transylvania region being well sheltered during cold air outbreak represents a more favorable region for wintering. From this point of view, we can consider that the Carpathian Mountains are a geographic barrier for wintering birds of prey.

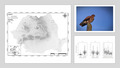

## 1 INTRODUCTION

Winter is a critical season in the life of most living organism inhabiting temperate and boreal latitudes (Askeyev et al., 2018; Hedenström, 2008; Speakman et al., 1991). In the most part of temperate zone and in all polar regions, winter is associated with high bioclimatic stress, which has led many animals to adapt for surviving (Blix, 2016). These adaptations were manifested by changing their behavior, getting into hibernation or deep sleep, or changing their distribution range, and migrating toward areas prone to milder winter climate.

Migration distance and strategies vary among species and populations, probably depending on multiple ecological factors (Hedenström, 2008). Some species migrate because their specific food disappears during winter, especially insectivorous species (La Sorte et al., 2015), others because they do not have the necessary physiological adaptations (Weber, 2009) while others try to move in more suitable areas where they can find an equilibrium for the energetic costs (Fort et al., 2013). Depending on the migration reasons, they can move thousands of kilometers (Battley et al., 2012), leaving their breeding grounds at the end of summer, migrating in milder climates areas, or, they can move with the cold weather to south, changing the wintering areas according to their climatic adaptations (Lemoine et al., 2016). Irrespective of the reasons for the migration, the trigger factors for these movements are represented by climatic variables (La Sorte et al., 2015).

Different species respond in a specific manner to the climatic variables. Some species could start the migration being pressed by the local climatic factors (Askeyev et al., 2018), but others are influenced by large‐scale mechanisms related to atmospheric circulation (Baltag et al., 2018). Often, these large‐scale indices seem to be valuable predictors of ecological processes than local climate (Hallett et al., 2004).

Up to now, several studies have identified among these factors—the North Atlantic Oscillation (NAO) (Hubálek & Capek, 2008; Stervander et al., 2008), Arctic Oscillations (AO) (Baltag et al., 2018), or local temperature (Carrascal et al., 2016)—as having clear influence on different populations. The NAO was found to influence mostly Western and Northwestern European bird population (Jonzén et al., 2002), while AO was identified only for Eastern or central‐eastern European as a driver of bird populations (Baltag, 2013; Baltag et al., 2018). The local temperature could influence the period length which they spend in a region or local movements in different habitats (Mazumdar et al., 2017). But, most of these studies were conducted on local or regional scale, in relatively similar geomorphological and climatic conditions. There are no clear distinctions in populations or areas which are influenced differently by continental indices, as NAO and AO, or even local conditions. At continental scale, based on the results of Trigo et al. (2002) and Schaefer et al. (2004), we can perceive that the propagation of NAO and AO effects is disrupted in the area of Carpathians, especially concerning the air temperature. Overall, in Central Europe, the NAO signal is stronger than AO signal (Dokulil et al., 2010) and the signal of NAO decreases toward the eastern part of the continent (Trigo et al., 2002). On the other side, recent studies have discovered that at regional scale, for the territory of Romania, the impact of NAO and AO on overall climate conditions—expressed by climatic water balance—is quite similar in intra‐ and extra‐Carpathians regions (Prăvălie et al., 2013). There is no information whether these climatic indices could influence the same species from different distribution ranges. Also, we do not know which is the main boundary for these influences, which could be marked by distance from the indices main action areas or by geographic barriers (mountains, large lakes, or others).

Mountain ranges could play an important role in separating climatic conditions and implicitly influences of the large‐scale atmospheric mechanisms between two or more regions through their role as barriers in air masses movements, as is the case with the Rockies Mountains from North America (Eidhammer et al., 2018). In Europe, there are some mountain ranges which are crossing the continent delimiting different climatic areas, as the Alps, but also, every mountain ridge imposes specific climatic features at regional and local scale (Barry, 1992). Carpathian Mountains are the second largest mountain range in Europe and have a major role in shaping the climate conditions in medium and lower Danube Basin, inducing a variety of local and regional conditions (Bâzâc, 1983). In Romania, the Carpathian Mountains separate the climatic conditions from eastern and western part of the country (Apostol & Sfîcă, 2013). However, up to now, there are no comparative studies to test their influence for bird distribution during winter when harsh weather could cause important changes in animal movements and survival.

In the present study, we aimed to explore the Common Buzzard number variation during the cold season and the climatic predictors of Common Buzzard wintering movements in relation to the possible influence of the Carpathian Mountains. Through our analyses, we are trying to answer the following main questions: (1) Is the number of Common Buzzard similar across the Romanian landscape? (2) Are the Common Buzzard wintering populations from east and west sides of the Carpathian Mountain influenced by the same factors? And finally (3) are the do Carpathian Mountains, the border between the NAO and AO, influences? To do so, we investigated the relationship between Common Buzzard numbers and continental and local climatic factors in two regions: one inside the Carpathian Arch and the other one outside, east of these large mountains, in Moldova region.

## 2 MATERIALS AND METHODS

### 2.1 Study area

**FIGURE 1:**
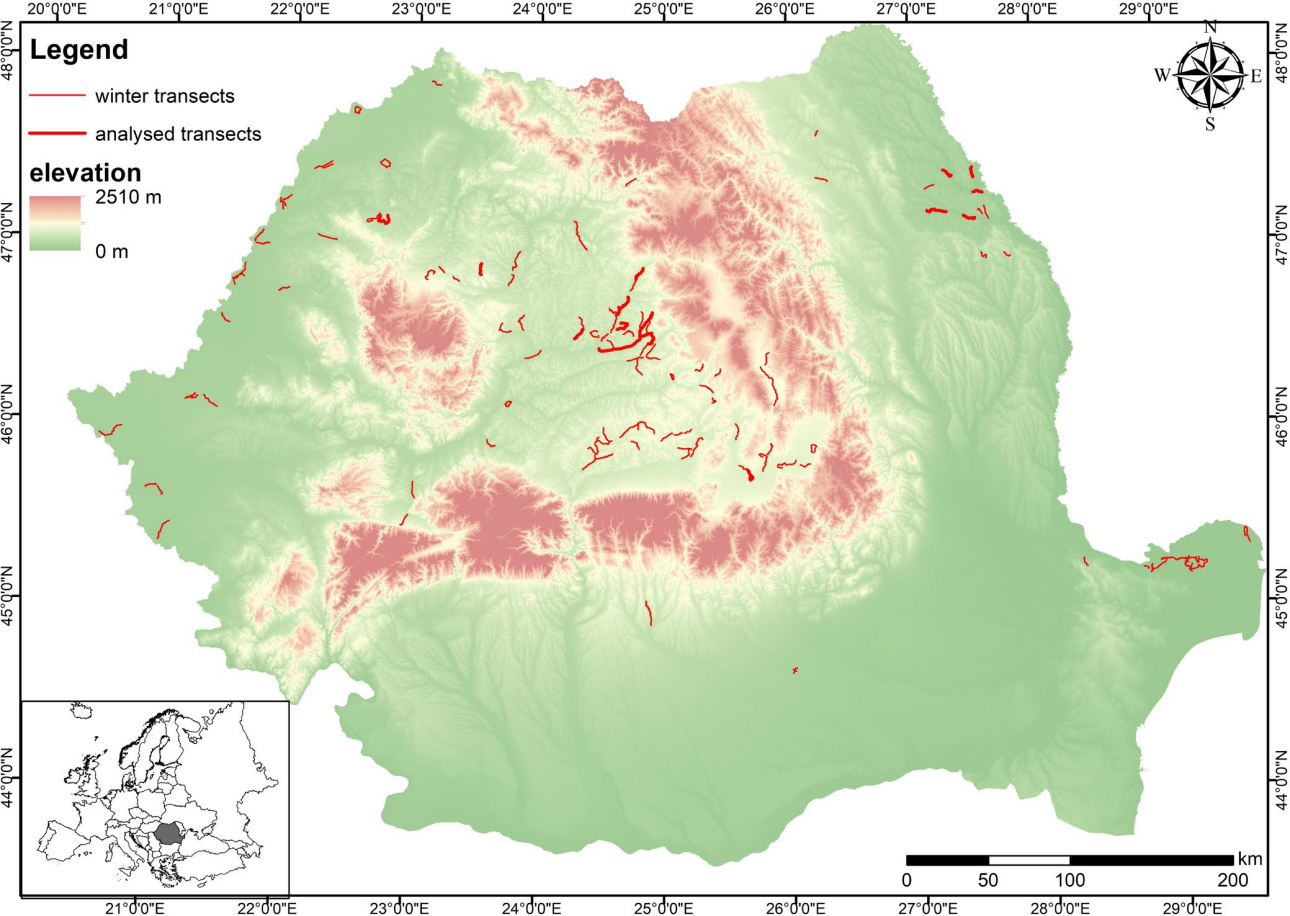
The distribution of Common Buzzard monitoring transects across Romania, during the 2006–2017 wintering seasons and the localization of our study area (black polygon) in Europe

The study area covers four regions from Romania, on both sides of the Carpathian Mountains (Figure 1). However, since we have long‐term data only for two areas (Transylvania and Moldova), we focused our analysis on these regions. The first one corresponds with Transylvania, inside the Carpathian Arch, a hilly area with many forest plots, mixed with agricultural land, pastures, localities, and river valleys. The second area is represented by North‐Eastern Romania, outside of the Carpathian Mountains, also a hilly area but with few forest plots, which are mostly concentrated in central and northern part of the region, as explained by Baltag et al. (2013). From physico‐geographical point of view, the main difference between the two regions is given by their position regarding the Carpathian Arch, which determines sheltering conditions for Transylvania during very cold spells originating from the northeast of the continent, while Moldova is more exposed to temperature oscillations during the same period of the year (Croitoru et al., 2018). This is reflected in annual temperature amplitude which is 2–3°C higher in extra‐Carpathian region than in intra‐Carpathian region (Sandu et al., 2008).

### 2.2 Data collection

The study method implies two data collection sessions each winter: in the first two weeks of December and in the second and third weeks of February. Observations take place along routes of at least 5 km length selected by participants with the help of the program coordinator. The route is covered on foot, and participants have to note down all birds of prey and weather conditions. On each transect, we collect data regarding the number of individuals per species, sex and age (if it is possible), distance to the observer (in 5 distance intervals): **1** (0–100 m); **2** (100–250 m); **3** (250–500 m); **4** (500–1,000 m); and **5** (>1,000 m), and the birds’ activity as per two behavioral categories (flying or perching). Also, the observers collect data on weather: visibility (in 5 categories: 1. up to 100 m, 2. up to 250 m, 3. up to 500 m, 4. up to 1,000 m, and 5. over 1,000 m), wind (in 4 categories: 0. no wind, 1. breeze, 2. moderate wind, and 3. strong wind), cloud cover (in 3 categories: 1. clear sky, 2. partly cloudy sky, and 3. completely covered with clouds), and precipitations (0. absent, 1. light/or drizzle, and 2. light snow). During the study period, 155 transects were covered but only 20% of these were repeated constantly during the 2006–2017 winter seasons. To avoid a high imputing of missing counts in our analysis, we select only constantly repeated transects. The routes are nonrandomly selected, mostly in open habitats where significant number of birds of prey are expected to occur.

### 2.3 Weather data

In order to understand the influence of weather on the variability of Common Buzzard numbers, we integrate into the GLMM analysis the continental indices (NAO and AO), air mass trajectories, and weather conditions in the study area. Daily values of NAO and AO indices have been downloaded from the Climate Prediction Center of NOAA (NOAA, 2018).

The daily data for NAO and AO were taken from the official site of Climate Prediction Center of NOAA (NOAA, 2018); the data were selected for each monitoring day and for 5 days previously. Our choice for 5‐day lag selection is mainly based on the fact that it was shown that some drivers of atmospheric circulation in Europe (e.g., NAO) have a mean persistence of 5 days. This persistence enables them to have an impact on the variability of weather elements over the continent (Keeley et al., 2009).

Considering that the migration can be triggered by changes in weather conditions in some remote areas, we have analyzed weather conditions for 5 days previously to the monitoring day from NCEP/NCAR for air temperature, precipitation amount, air pressure, and wind speed (Kalnay et al., 1996) in the source region of the air mass indicated by HYSPLIT analysis (HYbrid Single‐Particle Lagrangian Integrated Trajectory). This HYSPLIT technique from Stein et al. (2015) theoretically indicates in our study the easiest and therefore most probable path used by Common Buzzard individuals in their migratory route toward Romania. To strengthen the consistency of the results, this analysis was restricted to the highest number of individual observations. Therefore, only the backward trajectories for the upper third of number of individual observations were selected and the origins were displayed on maps as points. These points were grouped into 2 spatial clusters using k‐means clustering (*kmeans* function from *stats* package for R) for both regions.

Weather conditions for the monitoring day for the two regions were recorded from the nearby weather station (Târgu Mureş for Transylvania and Iaşi for Moldova) for air temperature (mean, maximum, and minimum), mean relative humidity, wind speed (maximum and minimum), precipitation amount, visibility (in km), and snow cover (in cm). The data were taken from Global Summary of the Day from NOAA (CPC, 1987), and well long‐term mean for 1961–2013 has been calculated from Romania Climate Dataset (Bîrsan and Dumitrescu, 2014).

### 2.4 Statistical analysis

The statistical differences in the Common Buzzard presence between regions and winter months were assessed using the Mann–Whitney test, and Kruskal–Wallis test for differences between winter seasons.

Multivariate analysis was conducted by applying a generalized linear mixed model (GLMM) approach, with stepwise backward elimination using 18 climatic parameters (Table 1). The number of Common Buzzard/linear km was selected as response variable for the GLMM approach, with a negative binomial distribution. Because the data on wintering Common Buzzard came from different transects, during two winter months and 11 years, we included “transect ID,” “month,” and “year” as random effects. All variables were scaled using *Z*‐score standardization. We excluded the least‐significant variables in a stepwise procedure, using Akaike's information criterion with correction for finite samples (AICc) value to select the best model (Tables 1 and 2). This model evaluation was done using the all possible subsets method with “MuMIn” package (Barton, 2020) for R statistical software v.3.2. To evaluate the model adequacy, the residuals versus fitted values and explanatory variables were plotted, but no distinct patterns were observed. The variance inflation factor (VIF) was used to check for multicollinearity, which was lower than 2. The final model was also tested for overdispersion using *pchisq* function (Kabacoff 2011). The relative importance of predictors was evaluated using *relaimpo* package (Gromping, 2006). We checked for spatial autocorrelation based on Moran's I test (Gittleman & Kot, 1990) with the “ape” package (Paradis et al., 2021). No significant spatial autocorrelation was found using Moran's I (*p* > .05 in all models). Calculations were made in R statistical software version 3.3 (R Core Team, 2013) with *glmmADMB* (Bolker et al., 2013) and *stats* (R Core Team, 2013) packages.

**TABLE 1:** Climatic data used for a generalized linear mixed model (GLMM) approach to determine its influence on the Common Buzzard's wintering abundance in Romania

Variable name	Variable abbreviation	Time window
Maximum Temperature	Tmax	t0
Minimum Temperature	Tmin	t0
Mean Temperature	Tmean	t0
Relative Humidity	RH	t0
Maximum wind speed	WindMax	t0
Mean wind speed	Wind Mean	t0
Visibility	Visibility	t0
Precipitation amount	Prec	t0
Snow depth	Snow	t0
Arctic Oscillation 0	AO0	t0
Arctic Oscillation 5	AO5	t5
North Atlantic Oscillation (same day)	NAO0	t0
North Atlantic Oscillation (5‐day previously)	NAO5	t5
Air temperature in source region	T_source	t5
Precipitation amount in source region	Prec_source	t5
Air pressure in source region	P_source	t5
Wind speed in source region	Wind_source	t5
Distance to the center of the air mass origins	Dist	t0

Note

For each variable provided there is a time window over which daily measurements are averaged ‐ t0 = the survey day, t5 = 5 days before the survey.

**TABLE 2:** Results of the model selection for the observed wintering Common Buzzards during 11 winter seasons (2006–2017), in Transylvania, Romania

Model	AICc	Log‐likelihood	Model weight
AO0 + Dist + NAO5*	2,196.4	−1,090.99	0.42
AO0 + NAO5*	2,198.1	−1,092.88	0.18
AO0 + Dist + NAO5* + Tmin	2,198.1	−1,090.78	0.18
AO + NAO5* + Tmin	2,200.0	−1,092.82	0.07
Dist + NAO5*	2,201.4	−1,094.57	0.03

Note

Models were fitted as GLMMs, with transect ID, month and year as random factors (intercepts). Five models constituting a 95% confidence set are presented, with Akaike's information criterion with correction for finite samples (AICc), model log‐likelihood, and relative model weight provided for each model.

We ranked models using Akaike's information criterion with correction for finite samples (AICc), using “MuMIn” package for R Statistical Software. For abbreviations, please refer Table 1.

* Significant variables.

## 3 RESULTS

**FIGURE 2:**
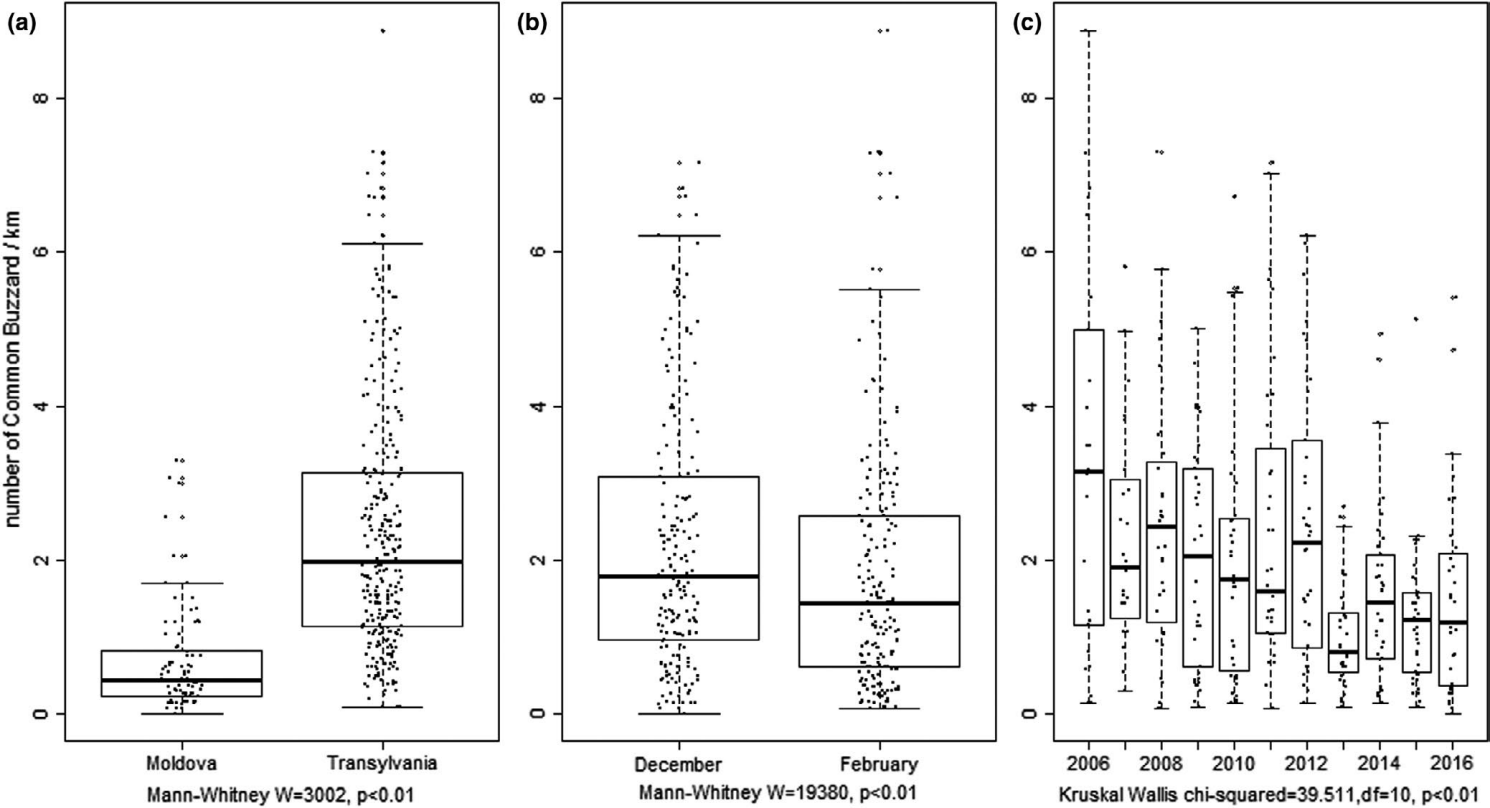
Comparison of Common Buzzard number/transects across the region (a), winter months (b), and wintering seasons (c) during December 2006–2016 wintering seasons

The Common Buzzard wintering population recorded in 2006–2017 period was significantly different in western (Transylvania region) and eastern (Moldova region) Carpathian Mountains (*W* = 3,002, *p* < .01). The higher numbers were recorded in Transylvania (mean = 2.378 individuals/km) and lower in Moldova (mean = 0.676 individuals/km). The Common Buzzard numbers also differ across the wintering months (*W* = 19,380, *p* = .01), being higher in December and lower in February, at the end of the wintering season (Figure 2). They vary also, across the 2006–2017 wintering seasons recording two peaks, in 2006–2007 winter and in 2012–2013 winter (chi‐squared = 39.11, *df *= 10, *p* < .01; Figure 2).

**FIGURE 3:**
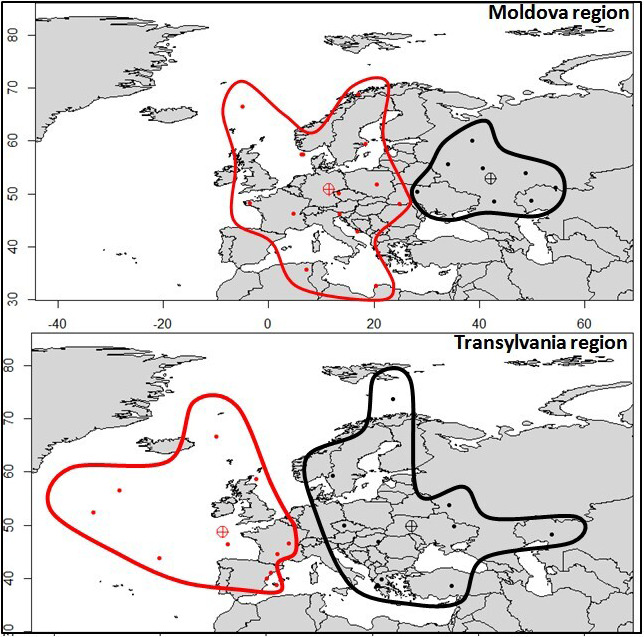
Air mass origins (represented by dots) for upper third cases of *Buteo buteo* individual observations in Moldova and Transylvania regions and the associated westerly (in red) and easterly (in black) clusters for both the regions

**FIGURE 4:**
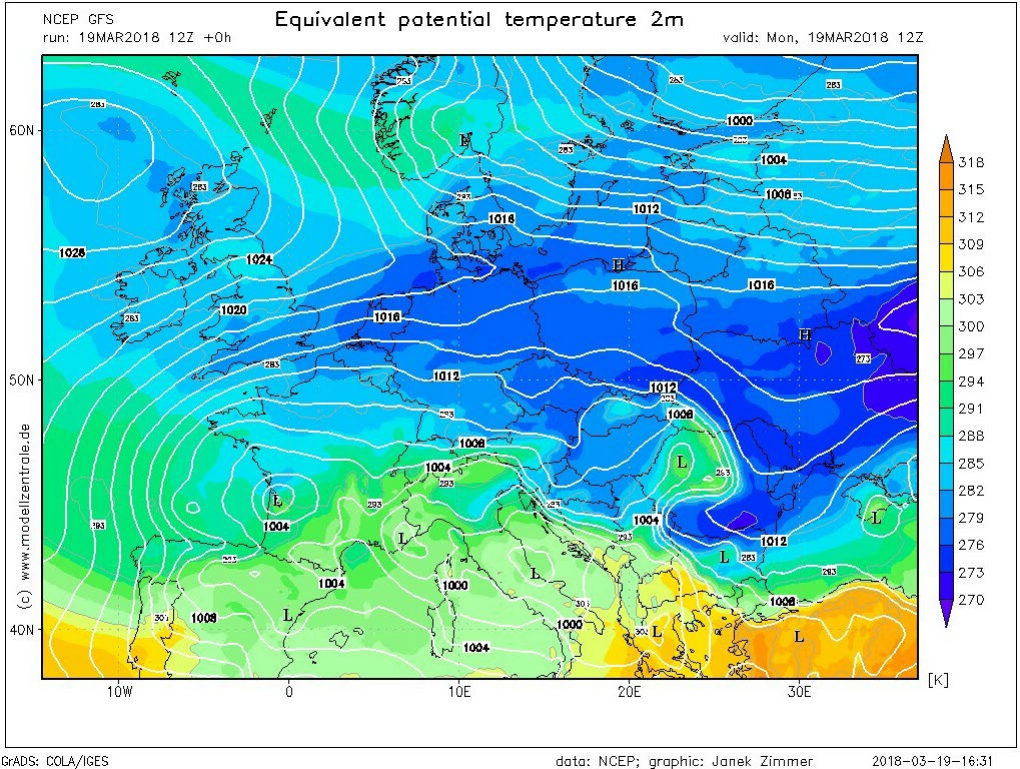
Distribution of air masses on continental scale during a severe cold air outbreak (March 2018) shows that due to the Carpathian sheltering the Transylvania region remains warmer/milder than any other parts of Europe during this type of winter episodes (Source: GFS via modellzentrale.de)

Using the HYSPLIT analyses, we observed that the air mass advections associated with the highest number of Common Buzzard observations in the two regions are located more westerly for Transylvania than for Moldova (Figure 3). As well, in Moldova region, the highest number of observation is associated with air mass advections routed in the eastern part of Europe and restricted to continental region, while in Transylvania region the air mass origins are shifted to the west (Figure 4). In other terms, these results show that there is a higher probability to have individuals with more westerly origins in Transylvania than in Moldova.

The wintering population of the Common Buzzard is consistently influenced by weather conditions in the study area. According to the GLMM analyses, the best selected model through stepwise backward procedure includes a low number of variables for both regions. The final models reveal that the Common Buzzard wintering populations are influenced differently in Moldova region (Table 2) than in Transylvania region (Table 3). The GLMM which analyses the influence on climatic variables on Common Buzzard numbers wintering East of the Carpathian Arch includes 4 variables for the final model, with AO as the only factor with a significant influence (*Z* = −2.87, *p* = .004; Table 4). For Transylvania, the final GLMM gathers 3 variables, but only NAO has a significant influence (*Z* = −2.17, *p* = .030; Table 5) on wintering Common Buzzards.

**TABLE 3:** Results of the model selection for the observed wintering Common Buzzards during 11 winter seasons (2006–2017), in Moldova, Romania

Model	AICc	Log‐likelihood	Model weight
WindMax + Visibility + AO0* + Prec_Source	431.6	−209.23	0.172
AO0* + WindMax	432.4	−210.82	0.112
AO0* + Prec_Source + Visibility	432.9	−209.92	0.087
AO0* + Visibility + WindMax	433.0	−208.73	0.086
AO0* + NAO0 + Visibility	433.4	−208.97	0.068

Note

Models were fitted as GLMMs, with surveyed squares and breeding seasons as random factors (intercepts). Five models constituting a 95% confidence set are presented, with Akaike's information criterion with correction for finite samples (AIC), model log‐likelihood, and relative model weight provided for each model.

We ranked models using Akaike's information criterion with correction for finite samples (AIC), using “MuMIn” package for R Statistical Software. For abbreviations, please refer Table 1.

* Significant variables.

**TABLE 4:** General linear mixed model (GLMM, R v.3.1.2) of factors influencing the Common Buzzard wintering numbers in Moldova region, Romania

Variable	Estimate	*SE*	*Z* value	*p*
Intercept	**1.543**	**0.094**	**16.38**	**<.001**
WindMax	−0.067	0.095	−0.71	.480
Visibility	−0.136	0.092	−1.48	.138
AO0	**−0.263**	**0.092**	**−2.87**	.**004**
Prec_Source	−0.086	0.090	−0.96	.339

Note

Model factors consist of weather‐related variables according to AIC selection. Significant *p*‐values (*p* < .05) are in bold. Total sample size covers 11 wintering seasons (2006–2017). For abbreviations, please refere Table 1.

**TABLE 5:** General linear mixed model (GLMM, R v.3.1.2) of factors influencing the Common Buzzard wintering numbers in Transylvania region, Romania

Variable	Estimate	*SE*	*Z* value	*p*
Intercept	**3.001**	**0.105**	**28.72**	**<.001**
NAO5	**−0.117**	**0.054**	**−2.17**	.**030**
Dist	−0.071	0.040	−1.79	.073
AO0	0.085	0.052	1.162	.106

Note

Model factors consist of weather‐related variables according to AIC selection. Significant *p*‐values (*p* < .05) are in bold. Total sample size covers 11 wintering seasons (2006–2017). For abbreviations, please refere Table 1).

Besides the continental indices, and despite the high number of weather variables taken to account, we did not identify any other variables that influence significantly the Common Buzzard in Romania across the wintering season.

## 4 DISCUSSIONS

The Common Buzzard wintering numbers vary on both sides of the Carpathian Mountains, being 3.5 times more abundant in Transylvania, compared with Moldova region. This difference could be induced by habitat structure (Baltag, 2013; Baltag et al., 2013), but also by a more stable climate, inside the Carpathian Arch due to its sheltering conditions (Apostol & Sfîcă, 2013) manifested especially during winter (Croitoru et al., 2018).

**FIGURE 5:**
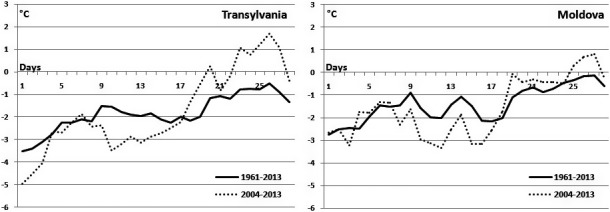
Air temperature evolution during February in Transylvania and Moldova for 1961–2013 and 2004–2013 derived from Romanian Climatic Dataset (Dumitrescu & Bîrsan, 2015)

Numbers vary also during the winter season, being higher in December, than in February. This difference could be related to their wintering movements, and probably in this month in Romania, there is an influx of individuals from more northern territories, individuals which are spreading after into other regions. December was also found the best wintering month, in term of Common Buzzard numbers, for Eastern Romania by other studies (Baltag et al., 2013, 2018). In February, Common Buzzard starts already migrating back to their nesting areas, leaving the wintering quarters. The wintering season variations could be determined by a decline in Common Buzzard population across Europe or by changes in migration strategies, possibly due to clime change. Common Buzzard could start the movements to the breeding places earlier than 10 years ago at the end of the winter. This strategy could ensure them better breeding places, because they will arrive earlier in the breeding grounds and will be able to occupy the best sites (Kjellén, 1994). However, the reports from Northern countries show an increase in Common Buzzard number for February besides the previous winter months, which means that they can occupy already the breeding grounds during this month (Väli et al., 2014). This hypothesis is supported also by the air temperature evolution during February in the recent period comparatively with the longer period of 1961–2013 (Figure 5) for Romania. This shows that the last period is characterized by a more abrupt increase in temperature. In our opinion is this more abrupt increase in air temperature at the end of February that encourages the northward migration of birds without stops in the region of Romania.

The results show that AO is the main large‐scale mechanism influencing the number of individuals observed, being present in the regression model for both regions. In winter, negative AO is associated with strong cold outbreaks in Europe (Croitoru et al., 2018; Thompson & Wallace, 2001; Wen et al., 2013), these conditions forcing the Common Buzzard individuals from eastern and northeastern Europe to move toward lower and warmer latitudes. In this line, a very important idea to be underlined is that a higher number of individuals is recorded when the atmospheric circulation pattern in Europe is from the east. However, for Moldova, the influence of AO is stronger (significant at *p* < .001) than for Transylvania for which the correlation is not significant. This difference is explained mostly by the fact that Moldova region is more exposed to cold air outbreaks associated with negative AO due to the lack of mountain barrier in the east (Apostol & Sfîcă, 2013; Croitoru et al., 2018). The influence of AO on Common Buzzard wintering population in Moldova has been demonstrated in another study (Baltag et al., 2013, 2018). The reconfirmation of AO influence is very important especially because the monitoring methods used in these two studies are different.

Normally, negative AO is associated with negative NAO, which is the dominant climate pattern in the north Atlantic region (Wallace & Gutzler, 1981). This climate pattern is demonstrated to have a considerably influence on ecological processes in Northern Hemisphere (Stenseth et al., 2003). Generally, negative NAO supposes an interruption of westerly flow in Europe, the atmospheric circulation being redirected mainly from the inner of the Eurasian continent toward the western Europe and Atlantic Ocean (Hurrell and Deser, 2009). The NAO has been found to affect numerous ecological systems and processes (Blenckner & Hillebrand, 2002; Westgarth‐Smith et al., 2012). The lower intensity of correlation for NAO in Transylvania could be given by an input of individuals in this region even during intense positive NAO, fact which can be suggested by the more westerly HYSPLIT clusters for this region. Also, being a mountainous region which is sheltered by the very cold air mass advection (Apostol & Sfîcă, 2013) another assumption is that a lot of individuals prefer to remain for long periods during the winter in this milder region.

Moreover, it is to mention that Transylvania region is very possible to remain warmer during severe cold outbreaks than any other neighboring regions of the continental Europe (Figure 4). Taking this aspect into consideration, the negative correlation with NAO supports rather the hypotheses of central European origins of individuals that are forced to migrate from the flat and more exposed regions by severe cold outbreaks (e.g., Poland, Baltic States, Belarus. and Eastern Ukraine) toward the more sheltered area of the Pannonian Basin and in Transylvania.

The main conclusion that could be driven from the analysis of meteoclimatic conditions consists in the different spatial origins of individuals observed in these two regions. While the high number of individuals in Moldova is related to their eastern and northeastern Europe origins, in Transylvania the large number of individuals observed is related to the more sheltered characteristics of the region attracting individuals from central‐eastern Europe. Also, since Transylvania region is well sheltered during cold air outbreaks, it represents a more favorable region for wintering. From this point of view, we can consider that Carpathian Mountains represent a geographic barrier for wintering Common Buzzard.

## CONFLICT OF INTEREST

None declared.

## AUTHOR CONTRIBUTIONS

**Emanuel Stefan Baltag:** Conceptualization (equal); data curation (equal); formal analysis (equal); funding acquisition (equal); investigation (equal); methodology (equal); resources (equal); software (equal); supervision (equal); validation (equal); visualization (equal); writing‐original draft (equal); writing‐review & editing (equal). **Istvan Kovacs:** Conceptualization (equal); data curation (equal); funding acquisition (equal); investigation (equal); methodology (equal); project administration (equal); resources (equal); supervision (equal); validation (equal); visualization (equal); writing‐review & editing (equal). **Lucian** **Sfîcă:** Conceptualization (equal); formal analysis (equal); investigation (equal); methodology (equal); software (equal); validation (equal); visualization (equal); writing‐review & editing (equal).

## Supporting information

Fig S1

## Data Availability

The data that support the findings of this study are available on Open Bird Maps Database: https://openbirdmaps.ro/.
