# Supplementary figures and images for: Common Buzzards wintering strategies as an effect of weather conditions and geographic barriers
Source: Ecol Evol. 2021 Jun 27;11(14):9697–706. doi: 10.1002/ece3.7793 (PMC8293765; doi:10.1002/ece3.7793)

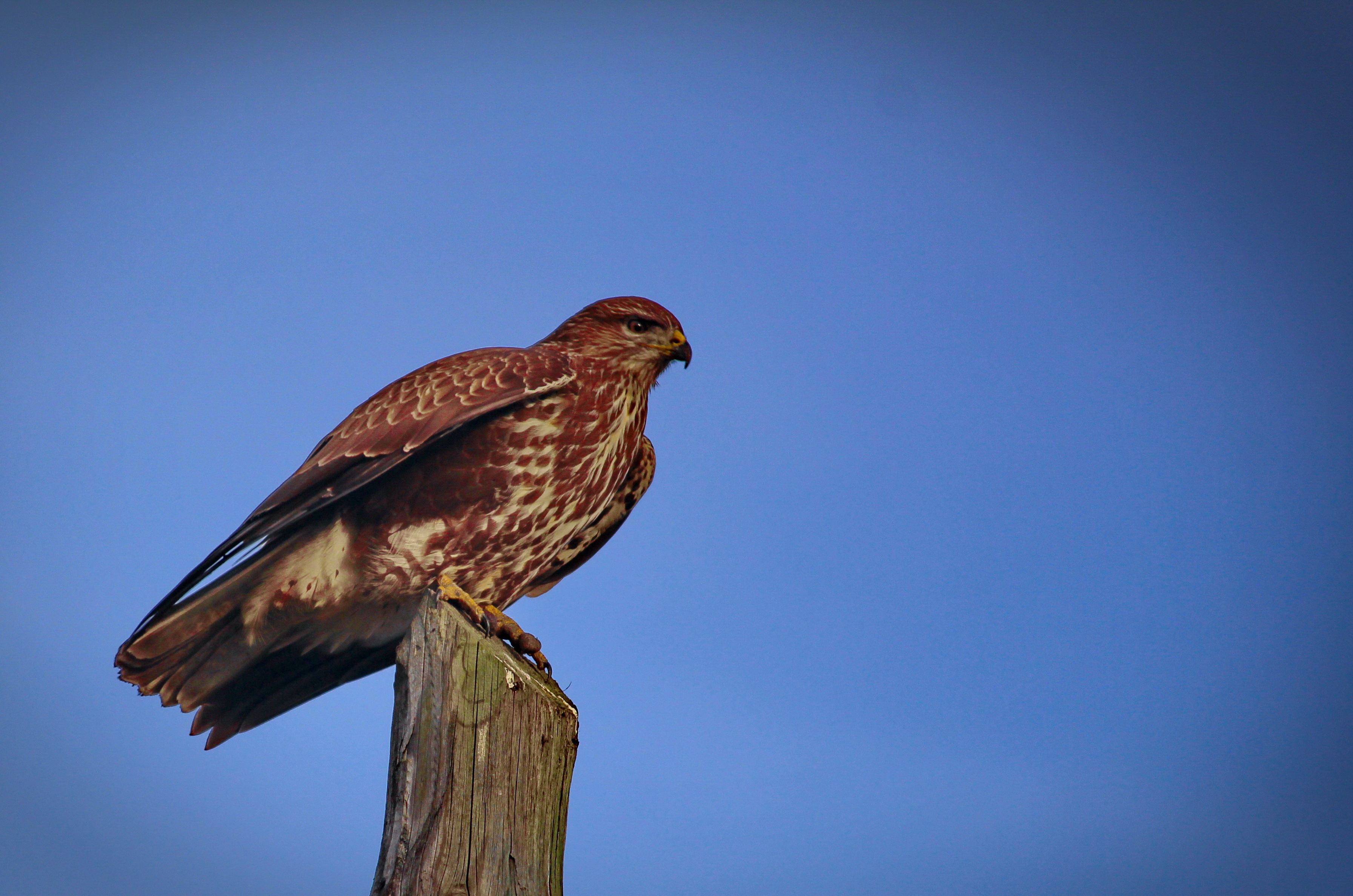

Supplement: Supplementary file 1 — Fig S1 [file ECE3-11-9697-s001.jpg]
